# Policy-maker attitudes to the ageing of the HIV cohort in Botswana

**DOI:** 10.1080/17290376.2017.1374879

**Published:** 2017-01-01

**Authors:** Kabo Matlho, Refelwetswe Lebelonyane, Tim Driscoll, Joel Negin

**Affiliations:** ^a^ PhD Candidate (Medicine) at School of Public Health, Sydney Medical School, University of Sydney, Sydney, Australia; ^b^ MD, MPH, is Principal Researcher and Coordinator of the Botswana Combination Prevention Project – Ministry of Health, Gaborone, Botswana; ^c^ MD, PhD, FAFOEM, FAFPHM, is a Professor of Epidemiology and Occupational Medicine at School of Public Health, Sydney Medical School, University of Sydney, Sydney, Australia; ^d^ MIA, PhD (The Main Supervisor), is the Associate Professor of International Public Health, Head of School, School of Public Health, Sydney Medical School, University of Sydney, Sydney, Australia

**Keywords:** ageing population, older adults, PLWH, HIV policy, vieillissement de la population, politique de VIH, plue adultes, PVVIH

## Abstract

*Background*: The roll out of antiretroviral therapy in Botswana, as in many countries with near universal access to treatment, has transformed HIV into a complex yet manageable chronic condition and has led to the emergence of a population aging with HIV. Although there has been some realization of this development at international level, no clear defined intervention strategy has been established in many highly affected countries. Therefore we explored attitudes of policy-makers and service providers towards HIV among older adults (50 years or older) in Botswana. *Methods*: We conducted qualitative face-to-face interviews with 15 consenting personnel from the Ministry of Health, medical practitioners and non-governmental organizations involved in the administration of medical services, planning, strategies and policies that govern social, physical and medical intervention aimed at people living with HIV and health in general. The Shiffman and Smith Framework of how health issues become a priority was used as a guide for our analysis. *Results*: Amidst an HIV prevalence of 25% among those aged 50–64 years, the respondents passively recognized the predicament posed by a population aging with HIV but exhibited a lack of comprehension and acknowledgement of the extent of the issue. An underlying persistent ageist stigma regarding sexual behaviour existed among a number of interviewees. Respondents also noted the lack of defined geriatric care within the provision of the national health care system. There seemed, however, to be a debate among the policy strategists and care providers as to whether the appropriate response should be specifically towards older adults living with HIV or rather to improve health services for older adults more generally. Respondents acknowledged that health systems in Botswana are still configured for individual diseases rather than coexisting chronic diseases even though it has become increasingly common for patients, particularly the aged, to have two or more medical conditions at the same time. *Conclusions*: HIV among older adults remains a low priority among policy-makers in Botswana but is at least now on the agenda. Action will require more concerted efforts to recognize HIV as a lifelong infection and putting greater emphasis on targeted care for older adults, focussing on multimorbidity.

## Résumé

Introduction: Le déploiement de la thérapie antirétrovirale auBotswana, comme dans nombreux pays avec près de l’accèsuniversel au traitement, a transformé le VIH une maladiechronique complexe mais facile à gérer et a conduit àl’émergence d’un vieillissement de la population avec le VIH.Bien qu’il y a eu quelques réalisation de cette évolution auniveau international, aucune stratégie d’intervention définisclairement n’établi dans de nombreux pays fortement touchés.C’est pourquoi nous avons exploré les attitudes des décideurset des fournisseurs de services pour le VIH chez les personnesâgées (50 ans ou plus) au Botswana. Méthodes: Nous avonsmené qualitatives entrevues face à face avec 15 personnelconsentant de la Ministère de la santé, les médecins et lesorganisations non gouvernementales participant àl’administration des services médicaux, planification, stratégieset les politiques qui régissent l’intervention sociale, physique etmédicale destiné aux personnes vivant avec le VIH et la santéen général. Le Shiffman et Smith cadre de comment lesquestions de santé deviennent une priorité a été utilisé commeun guide pour notre analyse. Résultats: Au milieu d’un VIHprévalence de 25 % chez ceux âgés de 50 à 64 ans,passivement, les intimés ont reconnu les difficultés posées parun vieillissement de la population avec le VIH maisprésentaient un manque de compréhension et reconnaissancede l’ampleur de la question. Un stigmate persistant âgistessous-jacente concernant le comportement sexuel existait chezun certain nombre de personnes interrogées. Répondants ontégalement signalé l’absence de soins gériatriques définis ausein de la fourniture du système national de soins de santé. Ilsemble, cependant, être un débat parmi les stratègespolitiques et les prestataires de soins quant à savoir si laréponse appropriée devrait être spécifiquement vers lesadultes âgés vivant avec le VIH ou plutôt d’améliorer lesservices de santé aux personnes âgées plus généralement.Répondants ont reconnu que les systèmes de santé auBotswana sont encore configurés pour maladies individuellesplutôt que des maladies chroniques pouvant coexister mêmes’il est devenu plus en plus courant pour les patients,particulièrement les personnes âgées, d’avoir deux ouplusieurs medical conditions en même temps. Conclusions: Le VIH chez les personnes âgées reste une faible priorité parmiles décideurs au Botswana mais est au moins maintenant àl’ordre du jour. Action nécessitera des efforts plus concertéspour reconnaître le VIH comme une infection permanente etmettant davantage l’accent sur les soins ciblés pour les aînés,en mettant l’accent sur la multimorbidité.

## Introduction

The policy considerations of ageing – economic security, health, disability and living conditions – are concerns throughout the world, but the nature of the problem differs considerably from continent to continent, between and within countries (Velkoff & Kowal, 2006). The advances of antiretroviral therapy (ART), in industrialized nations and many low- and middle-income nations, has transformed HIV into a manageable chronic condition leading to the emergence of a large population aging with the disease (Mills, Barnighausen, & Negin, 2012; Negin, Martiniuk, et al., 2012). This development has also ushered in a new challenge of co-managing a broad spectrum of morbidities associated with ageing in light of HIV. Despite this, aging with HIV is yet to achieve appropriate policy attention and focus.

The impact of the HIV epidemic on older people is shaped by the social, political, demographic, and economic circumstances in which they live (Report, 2010). HIV among older people in Africa is not something new, however, it has – until recently – been largely ignored (Morabia & Abel, 2006; Negin, Martiniuk, et al., 2012; Negin, Nemser, et al., 2012). Challenges posed by the ageing HIV epidemic are not insignificant (Nair, 2008). Although it has been widely reported that the older population makes up a small proportion of the overall population in most sub-Saharan African countries, recent reports and projections points to a considerable rise in the proportion of older people (Mahy, Autenrieth, Stanecki, & Wynd, 2014; Report, 2010). The number of people aged 60 and above in sub-Saharan Africa is projected to rise to over 67 million people by 2030 and almost quadruple the number documented in 2015 (46 million) to a record 161 million older persons in 2050 (Population_Division, 2016; Report, 2010).

The evidence points to a rise in the number of people living with HIV (PLWH) into older ages (as well as increased rates of new HIV infections within older persons) (Negin, Barnighausen, Lundgren, & Mills, 2012; Republic of Botswana Ministry of Heath, National AIDS Coordinating Agency, & Central Statistics Office, 2013). The primary cause, which has been widely noted in literature over the past decade, is that many HIV-positive people receiving appropriate care are living well into middle and old age (Report, 2010; Republic of Botswana Ministry of Heath et al., 2013; United Nations Development Programme, 2012). The quality of life of adults aging with HIV, however, is in question due to medical complications, poorer mental health, social isolation and stigmatization from health care providers and society at large (Barnett et al., 2012; Nair & Campbell, 2008).

Botswana is one of the countries most highly affected by HIV in the world. The total number of people aged 50 years and above, in Botswana, is 286,000 or 14% of the total national population (Republic of Botswana Ministry of Heath et al., 2013; Statistics Botswana, 2011).

The advances and roll out of ART in Botswana, as elsewhere, has transformed HIV into a manageable chronic condition leading to the emergence of a significant population aging with this disease (Farahani et al., 2014). Around 20% of the approximate 350,000 PLWH in Botswana are above the age of 50 years (Republic of Botswana Ministry of Heath et al., 2013). Nevertheless, Botswana, as do many sub-Saharan African countries, is unprepared to deal with this population ageing with HIV (Mahy et al., 2014; Ministry of Health Botswana, 2009; UNAIDS, 2014). While the main focus remains the universal roll out of ART to all PLWH, as documented in the Botswana Aids impacts survey 2013 at more than 80% coverage of eligible PLWH (Republic of Botswana Ministry of Heath et al., 2013), understanding the future needs for older people with regard to treatment for chronic and non-communicable diseases, medical complications, mental health and social isolation in older HIV-infected adults is limited (High et al., 2012; Negin, Barnighausen, et al., 2012; Negin, Nemser, et al., 2012; Negin et al., 2011; Work Group for HIV and Aging Consensus Project, 2012). There are a number of reasons to believe that traditional caring and social support mechanisms in Botswana are under increasing strain as a result (Viljoen, Spoelstra, Hemerik, & Molenaar, 2014).

Policy-makers’ attitudes are important for action (Jensen & Gaie, 2010; Jensen, Williams, Holyoak, & Shorter, 2013). Shiffman and Smith underline how specific health policies may be given a priority as a result of the confluence of a range of political factors, media attention and epidemiological changes in the population. Often, health, social and economic problems are a result of related rules, regulations, requirements and enforced behavioural pattern (Shiffman & Smith, 2007). The conjunction of the complexity of the problem and lack of governing policy can lead to policy stasis. The problems or process cannot be solved or expected to change without policy reform (Shiffman & Smith, 2007).

We therefore sought out policy-makers’ attitudes towards an understanding of HIV and ageing in Botswana, to better appreciate the barriers and recognize factors and facilitators that can lead to the development of an appropriate response to the challenge.

## Methods

### Participants

We conducted 15 one hour, face-to-face, semi-structured, in-depth interviews with consenting personnel from government and non-governmental organizations involved in the administration of medical services, care, strategies, planning and policies that govern social, physical and medical intervention aimed at PLWH and health in general in Botswana. The group consisted of six high-ranking civil servants directly involved with policy implementation from various departments within the government including the Ministry of Health and the National AIDS Coordinating Agency; four senior medical and nursing practitioners charged with HIV care at the HIV referral clinics in urban and rural areas; and four high ranking civil society representatives from three different non-governmental organizations directly involved with HIV care and management.

Purposive sampling (Suen, Huang, & Lee, 2014) was employed to identify the individuals. Each individual was approached via email and the interview proceeded if the individual consented. All 15 people approached consented to be interviewed. The interviewee were asked closely related questions however taking into consideration their position and work mandate, The interviews were carried out at most convenient time for the interviewee to maximize their capacity for concentration and patience. Interviews were conducted until saturation was reached.

### Interviews

A number of themes were raised during the interviews including: existing HIV services; existing aged care services; interviewee’s understanding of HIV among older adults; care for older PLWH in society; co-morbidities burden; and attitudes of care providers towards older PLWH. Twelve interviews were carried out in English and three were administered in Setswana. All interviews were recorded and then transcribed and translated into English where required.

### Data analysis

We used Shiffman and Smith’s framework (Shiffman & Smith, 2007) on determinants of political priority for global initiatives to guide our analysis. This framework has been used to examine why certain issues gain political prominence and others do not. The framework has been applied most prominently to maternal mortality and newborn survival (Shiffman, 2010, 2015).

While the framework was designed for use on a global level, we adapted it for use at the national level. The four categories of analysis we used are: actor power; ideas; political and health system contexts; and issue characteristics. The coverage of these four categories was: (1) the strength and the extent which the national leaders were involved in the initiative: ‘actor power’; (2) the nature of political and health contexts that inhibit or enhance support of the issue: ‘political and health system context’; (3) the organizations and political systems in place to depict the problems posed by HIV and ageing: ‘Ideas’; and (4) the power of some characteristics of the complexities surrounding HIV and ageing, to inspire action: ‘issue characterization’.

### Ethics

The Human Research Ethics Committee at the Ministry of Health in Botswana approved the project. Each interviewee consented to participate in the study. Confidentiality for interviewees was guaranteed to encourage more open discussion. Where direct quotes are provided, names are not attributed.

## Results

### Actor power

Interviewees noted the lack of high profile activists and community-based groups focused on the challenges posed by the ageing of persons affected by the HIV epidemic. Interviewees noted that in the early 2000s, the then President Festus Mogae personally galvanized the aggressive movement to scale up HIV prevention, treatment and care services. Several interviewees stated that the government is often compelled to take notice of emerging issues through the work of key leading figures and their NGOs but that in the case of HIV among older adults, no such leader had emerged.

I guess one could say there is no leading figure of any kind to shine the light on the issues pertaining to aged care in general. (#Interviewee 14)

One thing I can tell you is whenever there is a highly respected figure advocating for an issue; it tends to get attention from government officials, civil society and the general public. There is no individual or no organization that have stood up and said we want to look after the aged with HIV, the focus is always on the general care of all citizens, or if anything directed towards youth, I mean, there is even a Ministry and a cabinet minister dedicated specifically for youth agendas, but there is no such for the elderly. (#Interviewee10)

We (government) often work with NGOs, but there is no organization that have stood up and said we want to take the initiative and look after those aging with HIV, the focus is always generalized, or directed towards youth. (#Interviewee 3)

Interviewees underscored the importance of having a strong working relationship between government and civil society, as has been the case in the past on getting a number of health issues and social initiatives onto the political agenda. They emphasized that government often responds to civil society advocacy. Interviewees pointed that a lot of the HIV programmes coordinated by the National Aids Coordinating Agency were in fact NGO-driven or privately sponsored.

It shouldn’t just be about the government; NGOs have huge roles to play in this respect so that we can assist people who need a bit more care than ordinary. So far I don’t think there is any NGO looking at that cohort. I think, it is very important, however that this is addressed in the future. (#Interviewee 6)

NGOs have a role to play, and I do feel that, as a ministry, we have not used the NGOs enough to extend our hand in this matter but we should do that. (#Interviewee 2)

### Political and health system context

Geriatric care in Botswana is limited, however the interview revealed an emerging view and perception, among interviewees, that improved and well-targeted holistic multi-disciplinary delivery of health care to the elderly was essential. Interviewees pointed to the present void in health care programmes specifically designed to deal with the aged population as well as the lack of targeted aged care facilities and nursing homes despite the growing population of the frail elderly population in the society. Interviewees noted that most people believed that older people would be taken care of by their family as per established traditional and cultural norms.

We don’t have special programs that address the disease of the aged. There is no department that is specific for old people. We tend to focus more on the social protection, social services for the elderly, which is in fact dealt with under the Ministry of Local Government rather than Ministry of Health. (#Interviewee 2)

Geriatrics, in Botswana is very much under-developed. We don’t provide specific geriatric programs in our facilities, it is a field that we are yet to develop and I really think there is a need for that. (#Interviewee 1)

The aged are treated as part of the community, they receive health care services like any other Motswana would. And frequently those care needs are often left out to the family. (#Interviewee 6)

Interviewees pointed out that complications due to HIV treatment and management in Botswana have always been managed from the perspective of palliative care. Interviewees argued however that palliative care was too broad and encompassed many chronic diseases including HIV, diabetes, and cancers. They pointed out however, that while integrated services were essential, there was a greater need to define aged care in light of HIV specifically, which they stated often introduced a complex situation that demands provision of extra services such as community health care as well as dietary needs and psychosocial services, to supplement and compliment medical and clinical services provided.

Some of the interviewees referenced the successful rollout of ART as an achievement that has ushered a new challenge of dealing with HIV and ageing. This is a challenge they state is yet to be detailed and mapped out, but is most likely going to need integration and incorporation of services, ranging from medical services, community health care, and mental, spiritual and social health, to help manage.

We are one of the countries that have come a long way managing HIV and AIDS. We have a really successful antiretroviral (ARV) program you know and people don’t frequently die of AIDS anymore in Botswana; so clearly there is lot of people living with HIV in old age. (#Interviewee 11)

### Ideas

Interviewees disagreed about how the challenge of HIV among older adults should be framed. Some proposed that HIV services needed to be expanded to include older adults while others argued for comprehensive aged care services. Some lamented that health systems in Botswana seemed to be largely configured for individual diseases rather than co-existing chronic diseases.

Some people have been on ARVs for so long, at a late age, that presents a lot of challenges, pill burden, these patients could be on ARVs and concurrently taking other medications for other chronic illness, so unless we have comprehensive elderly care, it is very difficult to even talk about aged care and HIV because elderly care is non-existent. (#Interviewee 6)

The interviewees offered different explanations of what could be perceived as the existence of ageism in government provision of HIV services. Many argued that the main concern was not the age of the patient but rather, to make sure that all eligible PLWH were on medication and adhering to treatment to avoid issues like resistance and prevention of reinfection, while others underlined why special emphasis had to be given to the reproductive and more sexually active community.

Our HIV Core mandate has always been about general provision of ARVs and HIV prevention and services to the community, paying particular attention to the sexually active population, and reproductive population, which has always been defined as less than or equal to 49years. (#Interviewee 3)

There hasn’t been anything old aged specific for people living with HIV at policy level. When we roll out health services and improve HIV health services we often don’t single out any specific age, we just focus on HIV in general. (#Interviewee 2)

The problem of aging is bigger than HIV alone. Very soon, this country will see many people dying of non-communicable diseases than HIV. Of course when you have HIV you are special case but we really need to come up with broader policies addressing the older populations in general. (#Interviewee 5)

According to interviewees, the issue of HIV among older adults was also caught up amidst a framing of the role of older people. In Botswana, older people play critical roles as caregivers for the sick and guardians for orphaned grandchildren left behind. They point out that HIV has often taken the face of a woman because physically caring for the sick at any level of society has always been seen as a job reserved for women. At the height of the HIV epidemic, the majority of affected individuals were women, with some believing that was due to caring for their loved ones living with HIV or dying of AIDS. It has since been engraved not just in the mind of the community but rather in the approach and attitude of health care providers that the aged caught the infection from caring for their loved ones.

We often encourage the aged to test, and if they return a positive result, automatically assume they got it from caring for their probable infected loved ones. (#Interviewee 3)

A subconscious ‘moral’ question that is akin to passive ageism was also cited:

It is a scenario whereby, let’s say you have one kidney to give and there are two people who need a kidney, one is in their 20’s and the other is in their 60’s, whom should the lifesaving organ go to as first priority? The societies will often empathize more with the young. This is the mentality that is probably making people not to look to the aged. (#Interviewee 2)

Another theme that emerged is that older people are subjected to social isolation and stigmatization from health care providers and society at large. Older people are subconsciously not expected to be sexually active and therefore often not included as a specific focus group with regard to HIV prevention. There seems to be a subliminal tendency to turn a blind eye and detach sexual activity and thus not consider sexual transmission as a primary likely source of HIV infection among the elderly.

Older PLWH face institutionalized stigma, because society has a tendency to associate HIV with sex. This is exacerbated by the fact that a lot of our programs are only focused on the younger age cohorts. So it’s as if from aged 49 upwards we don’t want to talk about it. (#Interviewee 9)

People still stigmatize, and somehow health care provision adds to that. Even our condoms programs and marketing campaigns do not extend to older people. There seem to be this implied notion that the elderly are somehow not sexually active, and that is one major misconception. (#Interviewee 10)

Interviewer 4 claimed that in the past, Botswana used to have an extended family care and support system and now that fabric is dismantled. He alleged that migrations to urban areas to look for jobs by those that would in the past be staying home to look after parents has left the elderly all alone in the villages and rural areas to fend for themselves.

HIV aged patients are more likely to experience a lot of challenges in the society, not just health wise but socially. They are often less able to fend for themselves than when they were young able and working. (#Interviewee 5)

### Issue characterization

Some interviewees doubted the need to pay specific attention to this group suggesting that the issue of ageing and HIV had not been well argued.

The government acts out of need. So I suppose the question is; are they not getting services currently? Why do we think they need special services? You know, we need information that will impact the mind-set of policy makers to show [the] case that there is need for specialized services for the aged. (#Interviewee 10)

When you are on ARVs it’s OK, you just live like any normal person, make sure that you take your treatment and take ownership of your own health. So I don’t think old people living with HIV are treated any differently from everyone else above the age of 50. They are not even getting any special attention because when people get ARV’s they can live a normal live. (#Interviewee 13)

Some interviewers chronicled the existence of an underlying lack of a consensus on what to do about HIV among older adults, which they believe makes it hard to construct the issue as a priority when there is no clear set of activities warranting an urgent need for intervention.

At risk population such as Transgender, gays, and all the like, have in the past actually approached the government in need of services that will specifically cater for them, and the government’s response has always been, I have provided all health facilities and services to be accessed by everybody, why you? Now with the aged, I suppose the issue is also going to be; Why do we think they need special services, we need this information that will in a way convince those in authority that there is need for specialized care for the aged living with HIV. (#Interviewee 10)

Some interviewees also challenged the need for excessive focus on HIV in general, citing the success of the Botswana’s response.

HIV used to be a huge problem, a lot of people were dying and we all needed to come together to give people hope. (#Interviewee 4)

The interviewees acknowledged the evidence that there is an aging cohort, often subconsciously ignored, that has lived a long time with HIV, primarily due to successful ART. They alleged that the response to HIV appear to be largely focused on preserving the future generation (aiming for an HIV-free generation) or rather protecting the window of hope for the future generation, explained by the notable fixation on preventative programmes such as the prevention of mother to child transmission and prevention of spread of HIV geared toward the ‘sexually active’ (largely described as the ‘15-49’ age group) while the rest of the challenges are subconsciously believed to be catered for by the universal roll out of ARVs.

We don’t look at the age, at our health facilities nobody even notices the age transition; that perhaps as people get old, could mean other challenges. (#Interviewee 7)

There is no policy or anything within the guide lines of care that would guide one to say now you are moving from this age cohort to this age cohort, therefore one should look for other possible challenges. (#Interviewee 15)

Honestly there is nothing specific to aged care, unlike with paediatrics where there is a defined policy and the health care worker is aware of the change in the provision of care as the children grow up. But when it comes to adults, there is nothing to signify change in age; no one notices the aged in the context of HIV and care really. The concern would rather be on medication burden, may be due to individual being on medication for a long time etc. (#Interviewee 4)

The interviewees pointed that even though Botswana’s health management appeared to recognize HIV as a public health challenge, its focused intervention often put greater emphasis on HIV in the context of a sexually transmitted infection rather than a chronic lifelong infection, thus explaining the lack of specialized care to deal with the changing dynamics HIV presents as a manageable infection that, however, presents a lot of clinical challenges due to the weakening of the immune system. They assert that an implied perception that old age is synonymous with retirement from all sexual activities is rampant in a lot of communities in Botswana. This societal assumption that as people age, particularly women, their interest in sexual intercourse somewhat diminishes is however unsubstantiated.

When menopause sets in, there are less challenges’, things start to quieten, so I don’t know about the men but that is the case with women, but yes, there may be a rather different scenario with men. (#Interviewee 2)

Batswana are so complacent, we still have a long way to go, there are some elderly men above 50, who knows they are HIV positive but will still go have sex with young ladies. There are those that get into extramarital affairs and intergenerational relations, often termed ‘small house’, concubine so to speak. So all this needs to be addressed. (#Interviewee 9)

## Discussion

HIV among older adults in Botswana has not become a priority issue in the national HIV response, even though there has been some emerging signs that the epidemic is increasingly involving older people, therefore ushering in distinct HIV management challenges (Republic of Botswana Ministry of Heath et al., 2013; United Nations Development Programme, 2012). Shiffman and Smith’s framework stresses the importance of actors taking advantage of policy windows to influence decision-makers (Shiffman & Smith, 2007). The issue of HIV and aging is seldom discussed in policy circles and, when it is, it is blended within the context of geriatrics and gerontology.

Furthermore, there seems to be confusion in understanding how to conceptualize the issue with many respondents involved in shaping policy framing it largely as stemming from a lack of a holistic geriatric care while others agree with the status quo, arguing that the solution to the problem posed by aging in light of HIV is in fact provided within the general HIV care strategy. This reflects the general perception and lack of understanding at the global level of the health implications of living in older age with HIV (Mahy et al., 2014; Mills et al., 2012; Morabia & Abel, 2006; Negin & Cumming, 2010; Negin, Martiniuk, et al., 2012; Negin, Nemser, et al., 2012; Negin et al., 2011; UNAIDS, 2014; Viljoen et al., 2014).

The policy-makers’ perception of dealing with HIV in older people points to what remains the general focus of Botswana HIV intervention; the application of effective ART for HIV, which has allowed many infected persons to live to an older age. However institutionalized ageist connotations regarding sexual interaction by older people may also yield increased HIV incidence within this cohort as they are least likely to practice safe sex, a risk exacerbated by the late-life changes in the reproductive tract and immune system that may enhance susceptibility to HIV acquisition in older people (Negin, Nemser, et al., 2012).

The major focus for the Botswana government remains treatment and prevention aimed at the younger population rather than the broader challenge that comes about as a consequence of the success of ART based intervention. Too little is being done to address the impact of HIV on older people in Botswana, as it is not widely seen as of paramount importance. Lack of policy geared toward the issue arguably stems from the policy-maker’s disengagement and lack of comprehension of the magnitude of the problem and unique complications posed by the aging epidemic and the unfavourable outcome that could stem from the lack of this provision and preparedness in dealing with the aged cohort of PLWH.

There is a need to revise the HIV public health policy from primarily being focused on the young generation to reflecting the aging epidemic and the public health challenges it poses if Botswana is to ever achieve the ambitious goal of zero new infection (UNAIDS, 2011, 2014; Viljoen et al., 2014). Although the UNAIDS 2014 report, suggest that people aged 50 years and older often exhibit many of the risk behaviours also found among younger people (UNAIDS, 2014), the overwhelming support from interviewees seemed to be geared towards an establishment of a general aged care as opposed to exclusively focusing the attention on provision of specialized HIV aged care. Intergenerational relations are rife in Botswana and neglecting the elderly in the HIV fight is counterproductive and could result in regression of the progress made thus far (Jensen & Gaie, 2010).

There is a realization that the stigma and fear that used to accompany living with HIV has subsided in Botswana and with that there has been a diminished drive for community led change and mobilization to initiate political and hence government support. There are no identified national or community leaders that champion the cause to highlight the need for specialized HIV aged care on the national agenda. Few national or regional HIV prevention campaigns explicitly target older people. Aging with HIV is completely ignored in the Millennium Development Goals set for eradicating HIV in Botswana despite compelling evidence that mortality due to HIV has fallen as well as taking in to consideration the longevity of the epidemic (Farahani et al., 2014; The Government of Botswana, 2010).

In other studies examining policy priority for action, it was found that some health campaigns are easier to promote than others because an issue is perceived as more detrimental and the economic consequences of inaction are clearer (Shiffman, 2010, 2015). Global mental health is one initiative that has used the Shiffman and Smith framework to demonstrate that while some significant strides have been made, mental health still faces major challenges in establishing itself as a global initiative with meaningful political priority (Tomlinson Mark, 2012). In the case of HIV and aging it has been difficult to develop a common construct that can be promoted. The fact that the statistics about older adults are not well-documented means there is under-reporting as well as under-representation of the magnitude and the scale of the problem. This is unfortunate as such information is what catches the eye of politicians; it is most likely that not having a relevant or rather defined geriatric system and older adults’ representatives lead to underestimation of the significance of care needed for older PLWH. There are no credible measures that demonstrate the severity of the problem enough to alarm politicians or decision-makers.

### Study limitations

Although this study was effective in exploring the prevalent issues and views regarding aging and HIV management in Botswana, There were a limited number of primary data sources that could contribute to the study. Having 15 interviewees potentially left room for partiality.

## Conclusions

Differing professional views on HIV and aging, and disparities over interventions in Botswana, severely constrains, innovative, strategic and management capacities. There is a pre-eminent requisite for national and political recognition of the problem. Furthermore, the lack of political and community-based advocacy initiatives for aging and HIV makes the challenges posed by HIV and aging difficult to gauge and recommend proper and focused interventions. There is a clear exclusion of older adults living with HIV from major Legislation, public and HIV health policy initiatives and programmes in Botswana even though it is apparent that current effective treatment has prolonged the life of PLWH and likely increased life expectancy. This oversight could pose a serious setback in the fight against this epidemic. Left unaddressed, however, generations of older adults living with HIV will lack the supports they need to age in good health.
